# Correlation of PTC Taste Status with Fungiform Papillae Count and Body Mass Index in Smokers and Non-Smokers of Eastern Province, Saudi Arabia

**DOI:** 10.3390/ijerph17165792

**Published:** 2020-08-10

**Authors:** Asim Mustafa Khan, Badr Al-Jandan, Amr Bugshan, Khalid Al-Juaid, Saqib Ali, Reshma Veerankutty Jameela, Nasser Al Madan, Alaa BuHulaiga

**Affiliations:** Department of Biomedical Dental Sciences, College of Dentistry, Imam Abdulrahman Bin Faisal University, Dammam 34212, Saudi Arabia; baljandan@iau.edu.sa (B.A.-J.); abugshan@iau.edu.sa (A.B.); 2150004543@iau.edu.sa (K.A.-J.); samali@iau.edu.sa (S.A.); rvbeevi@iau.edu.sa (R.V.J.); 2120001722@iau.edu.sa (N.A.M.); 2130007263@uod.edu.sa (A.B.)

**Keywords:** body mass index, obesity, propylthiouracil, smoking, taste, tongue

## Abstract

Phenylthiocarbamide (PTC) is a bitter compound that is similar in taste to the polyphenols present in most vegetables and fruits. The human taste response towards this compound influences dietary preference, which has a bearing on an individual’s body mass index (BMI). Another factor that influences taste perception is fungiform papillae count. This, in turn, is governed by genetic factors or deleterious habits such as smoking. Establishing a link between all the above factors could lead to a wider understanding of obesity, which is a global health issue. PTC taste response, BMI, and fungiform papillae were recorded and statistically analyzed between two groups—smokers and nonsmokers. There was no statistically significant difference between smokers and nonsmokers with regard to PTC tasting ability. However, there was a significant inverse relationship between BMI and PTC tasting ability and fungiform papillae count both in smokers and nonsmokers. Thus, it can be inferred that as BMI increases, there is a lower likelihood of experiencing the bitter taste of PTC. Additionally, the ability to taste PTC decreases with diminishing numbers of fungiform papillae. Smoking does not affect bitter PTC tasting ability despite negatively affecting fungiform papillae count.

## 1. Introduction

Phenylthiocarbamide (PTC) is an unpleasant organic compound incidentally found by Fox. It has been extensively applied in several studies related to taste perception [1]. It is also called phenythiourea because of the presence of a phenyl ring and sulphur, forming an organosulfur compound responsible for its bitter taste [2]. This organosulfur compound is chemically similar to glucosinolate compounds that are present in cruciferous vegetables and impart a bitter taste [2]. PTC taste can only be detected by individuals who have a single dominant allele or two dominant alleles. Those who cannot detect its taste are those who have two recessive alleles in their gene pool [2].

The World Health Organization has reported obesity as a major public health issue worldwide due to the complications and mortality associated with it. Obesity has a multifactorial etiology but is mostly under the influence of hereditary factors and lifestyle. Taste has a key role in the selection of one’s diet, which is an integral part of one’s lifestyle [3]. This dietary behavior is a major factor affecting overweight and obese individuals [4]. In Saudi Arabia, a 2019 study based on anthropometric measurements among children aged 9 to 11 years and adolescents aged above 11 years stated an incidence of overweight and obese of 30.9% and 34.7%, respectively [5]. In Al-Kharj, Saudi Arabia, the prevalence rate of obesity and overweight is reported to be at 54.3% [6]. Al-Quwaidhi estimated that the future prevalence trend of adult obesity would rise to 41% in males and 21–78% among females in Saudi Arabia by 2022 [7]. The body mass index (BMI) devised by Adolphe Quetelet is an excellent tool that is used to evaluate an individual’s physical status in terms of weight and height and is easy to work with [8] and, therefore, is applied in this study to assess the participants’ BMI. 

Another major health issue in Saudi Arabia is the high prevalence (70.7–48%) of smoking seen in young adults, the majority of which being males [9]. Smoking is also a major cause of changes in taste functionality [10]. The sense of taste predominantly guides an individual’s taste preference and eating habits [11]. The sensory modality of taste helps to distinguish between various food substances, thus influencing food choice and consequently the nutritional status and health of the individual [12].

The tongue plays a major role in an individual’s dietary choice, as it is the first organ that comes into contact with food [13]. The surface of the tongue is embedded with numerous papillae, among which are the fungiform papillae, which morphologically resemble the shape of mushrooms. They are pink in color and are situated on the anterior two-thirds of the dorsum of the tongue. Their maximum concentration is on the tongue tip, with a gradual reduction in numbers in the posterior parts of the tongue. Taste buds contain taste receptor cells situated in fungiform papillae and are innervated by chorda tympani [1]. The taste receptor cells have protrusions called microvilli on the superior surface of the bud, which allow the cells to interact with taste stimuli present in the saliva [14]. Research data have shown that the number of fungiform papillae can be used as an objective indicator to evaluate taste perception [1,4,10,15,16]. However, the relationship between fungiform papillae and BMI is still not clear. Additionally, there are no sufficient data regarding the relationship between PTC tasting and BMI in the Saudi population. Whether this relationship is also influenced by factors such as the number of fungiform papillae and smoking habits was explored.

## 2. Materials and Methods

After obtaining ethical approval from the Institutional Review Board of College of Dentistry at Imam Abdulrahman Bin Faisal University, this study was conducted at the associated dental hospital between November 2019 and February 2020. The study design conformed to the Declaration of Helsinki for Medical Research involving human subjects.

Our study sample consisted of 200 participants who were from the regular outpatients at the dental hospital at Imam Abdulrahman Bin Faisal University. Subjects were divided into a study and a control group consisting of 100 participants each. The experimental group included a total of 85 males and 15 females, whereas the control group contained 9 females and 91 males. 

All the subjects in both groups were between the ages of 25 and 45 years, because fungiform papillae are found to be degenerated beyond the age of 45 years [17]. The study group included subjects who had been cigarette smokers for a minimum duration of 6 to 7 years, as the legal age to smoke in Saudi Arabia is 18 years [18]. This would also ensure that a considerable duration had elapsed to demonstrate any changes due to smoking. Participants who used various kinds of electronic cigarettes or vapers were not included in this study. Controls included nonsmoking subjects similar to the study group for age and gender. Those who had quit smoking were not included in this study. 

Subjects with a history of otolaryngologic diseases, chronic rhinitis, and sinusitis were excluded from the study. Subjects with tongue conditions such as ankyloglossia, geographic tongue, median rhomboid glossitis, or any other atrophy or malignancy of tongue were excluded from the study, as these would be obstructive to the counting of fungiform papillae. Subjects with a drug history of antibiotics, anti-amoebicides, antihelmintics, local anesthetics, clofibrate, chlorpheniramine maleate, diuretics, antidiabetic drugs, calcium channel blockers, or anti-Parkinson’s disease medications were eliminated from the study, as the above drugs are known to result in taste alterations [19].

To quantify smoking habits, smoking was calculated in terms of packs per year (packs/year). This was based on the following simple formulae: 

Packs of cigarettes smoked per day X number of years smoked = packs/year

or

Number of cigarettes smoked per day X number of years smoked ÷ 20. A divisor of 20 was selected because a pack of cigarettes contains 20 cigarettes according to most of the international manufacturing standards [20,21]. 

Taste strips:

PTC (phenylthiocarbamide) taste test paper strips along with control paper strips manufactured by Precision Laboratories (Cottonwood, USA) were used in this study. The taste strips were 1.875 (length) × 0.25” (width) and predominantly composed of cellulose. Each taste strip contains 20 µg of the PTC chemical, whereas the control strip was devoid of any chemical substance. All strips came in a vial of 100 s, with a shelf life of 2 years. 

Procedure:

Each participating subject underwent a thorough medical history evaluation and an oral examination after obtaining informed consent. This was succeeded by a BMI assessment by noting down the height and weight of the subject using a digital weighing scale. All the subjects were instructed not to drink, eat, or smoke for an hour prior to the beginning of the test. 

This was followed by training the subject to respond when a taste strip was placed on the anterior two-thirds of the dorsum of the tongue. The subjects were asked to respond with a “yes” or “no” to indicate whether they detected any sensation of taste. The subjects’ tongues were cleaned with cotton gauze before placement of the test strips each time. The PTC and control strips were used on subjects of both the experimental and control groups so that responses from both groups could be recorded. This was performed in a blind manner to ensure that none of the participants were aware of the kind of test strip used during each attempt. After this, the subjects were asked to protrude their tongue in a relaxed position and a photograph of the tongue was taken using a digital camera with flash. These photographs were then used to calculate the fungiform papillae count using image analysis software. The image was overlapped with grid boxes of 1 cm^2^, and papillae in each were counted and averaged to give the mean fungiform papillae count (FPAP) for each subject. 

The data obtained were compiled and subjected to statistical evaluation using Pearson’s correlation, descriptive statistics, and an independent samples *t*-test with the aid of SPSS for Windows. 

## 3. Results

The study sample consisted of 200 subjects. The experimental group included 100 subjects who were smokers, and 100 nonsmoking subjects comprised the control group. All subjects were in an age range from 25 to 45 years.

### 3.1. Age

**Figure 1:**
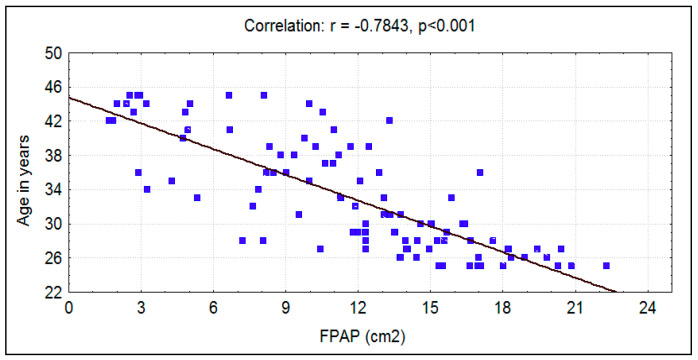
Correlation between age in years with fungiform papillae count (FPAP) (per cm^2^) in smokers.

**Figure 2:**
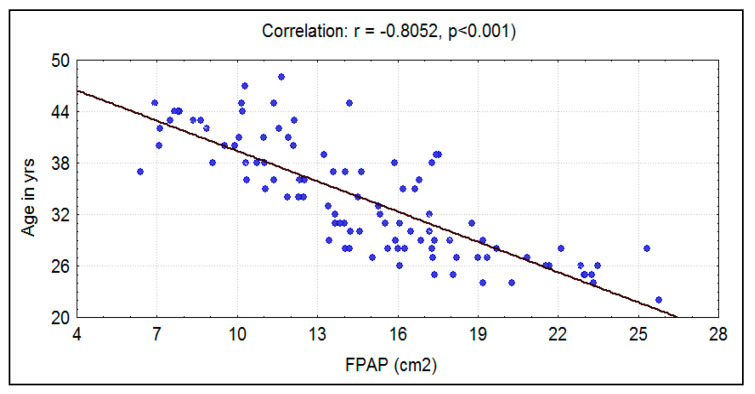
Correlation between age in years with fungiform papillae count (FPAP) (per cm^2^) in nonsmokers.

A mean age of 33.03 ± 6.47 was recorded in the experimental group, and an age of 33.8 ± 6.65 was recorded in the control group. There was no significant difference (*p* = 0.407) between the two groups in relation to age. However, a significant strong negative correlation existed between age and fungiform papillae count in both groups (Table 1) (Figure 1 and Figure 2). 

**Figure 3:**
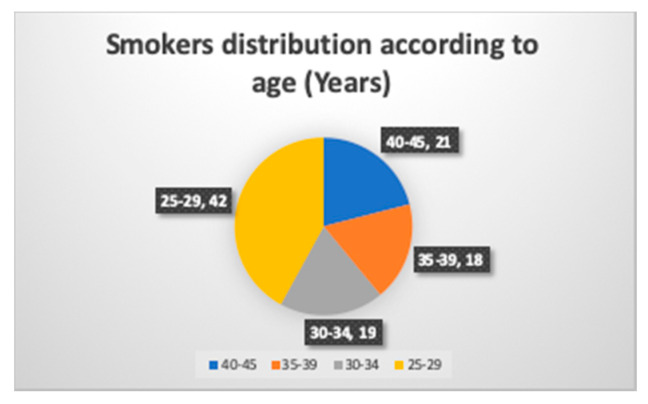
Age wise distribution of smokers.

**Figure 4:**
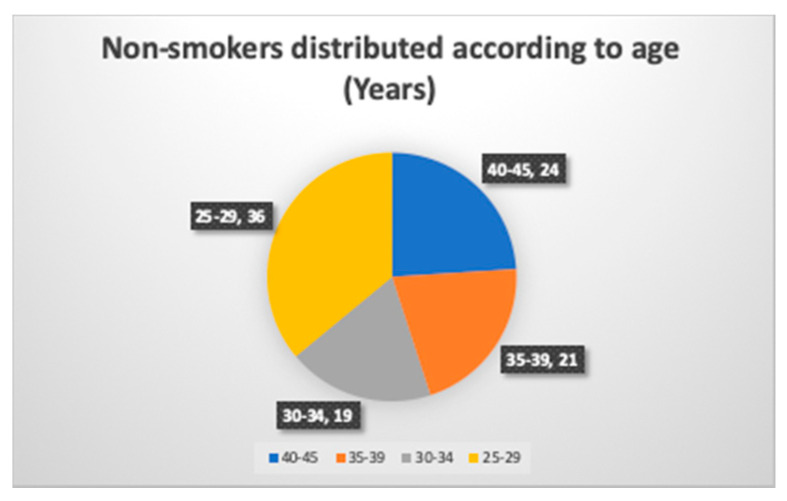
Age wise distribution of smokers.

There were 21 smokers and 24 nonsmokers in the age range of 40 to 45 years, and two in each group gave a positive response to the PTC taste test (Figure 3 and Figure 4). One of them, a 43 year old male smoker, had a BMI of 30.4 and a fungiform papillae count of 10.55 per cm^2^. The other smoking subject was a 45 year old male with a BMI of 27.11 and a fungiform papillae count of 8.11 per cm^2^. On the other hand, in the nonsmokers, a 40 year old male with a BMI of 31.3 and a fungiform papillae count of 9.52 per cm^2^ could detect bitterness in the PTC taste strip. Another nonsmoker who could detect bitterness in the PTC strip was a 43 year old male with a BMI of 29.4 and a fungiform papillae count of 12.14 cm^2^.

### 3.2. BMI

**Figure 5:**
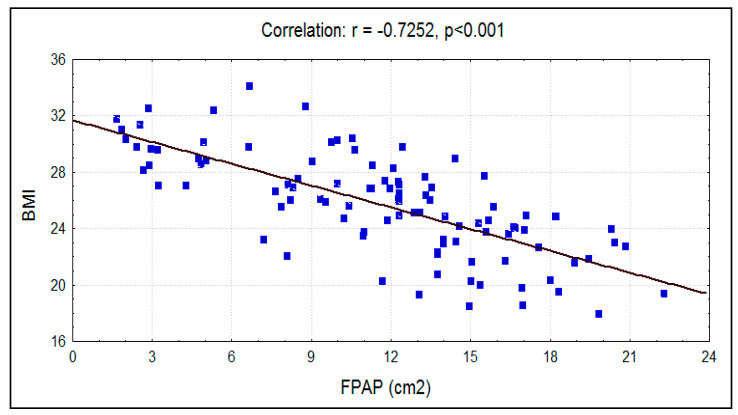
Correlation between BMI with FPAP (per cm^2^) in smokers.

**Figure 6:**
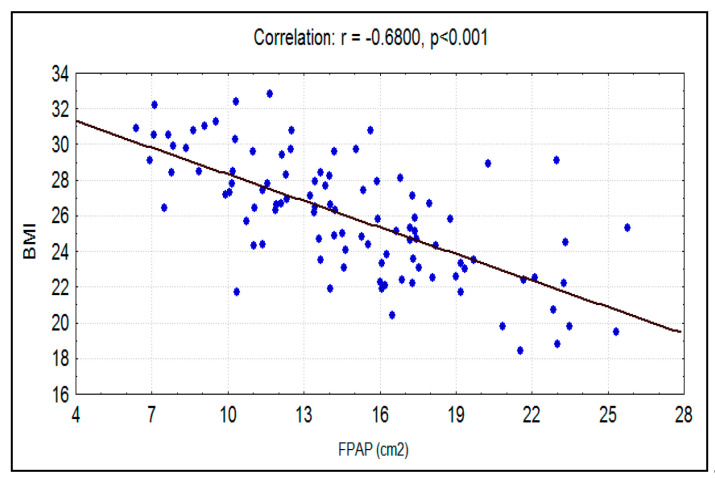
Correlation between BMI with FPAP (per cm^2^) in nonsmokers.

The mean BMI for smokers was 25.67 ± 3.59, whereas in nonsmokers, the mean BMI was 25.82 ± 3.45. There was no significant difference (*p* = 0.548) between the means of BMI between the two groups, but the Pearson correlation between BMI and fungiform papillae count showed a significant strong negative correlation between the two factors in both the experimental group and the control group (Figure 5 and Figure 6). Overall, the mean BMIs of the subjects of the entire sample who positively and negatively responded to PTC were 24.25 ± 3.11 and 26.62 ± 3.35, respectively. A significant difference (*p* < 0.001) between them was recorded using the independent *t*-test (Table 2).

The experimental group consisted of 9 obese subjects. The maximum BMI recorded in a 41 year old male was 34.08. He had a fungiform papillae count of 6.69 per cm^2^ and responded negatively to PTC. However, a 43 year old male in the experimental group responded positively to PTC and reported a BMI of 30.4 and a fungiform papillae count of 10.55 per cm^2^. The difference in response to PTC in the above two subjects could be attributed to the smoking factor, the former recording smoking at 9 packs per year and the latter reporting 3.75 packs per year. This shows that smoking has a bearing on fungiform papillae count. In the control group, the maximum BMI recorded was 32.8 in a 45 year old male. He could not taste PTC and reported a fungiform papillae count of 11.65 per cm^2^. In total, the control group had 12 obese subjects, of which only two could detect PTC taste. One of them was 36 year old male with a BMI of 30.8 and a fungiform papillae count of 12.52 per cm^2^. The other obese subject in the control group who tasted PTC was a 40 year old male with a BMI of 31.3 and a fungiform papillae count of 9.52 per cm^2^.

### 3.3. Fungiform Papillae Count

**Figure 7:**
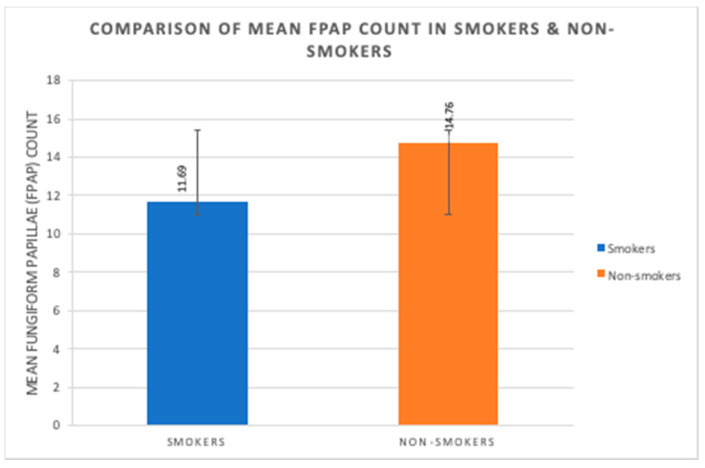
Comparison of mean fungiform papillae count (FPAP) (per cm^2^) of smokers and nonsmokers.

**Figure 8:**
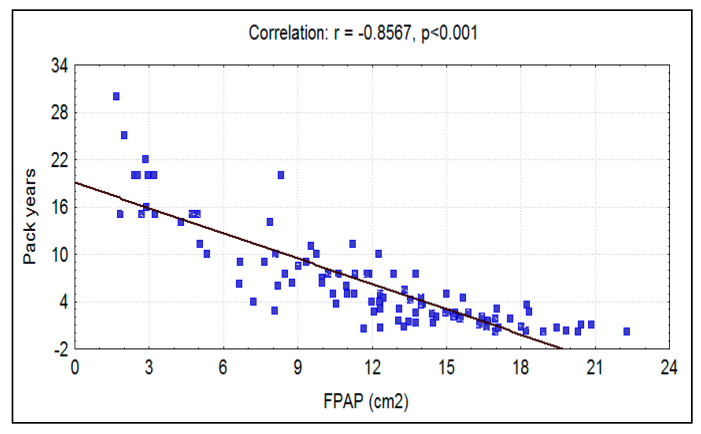
Correlation between pack-years with FPAP (per cm^2^) in smokers.

The mean fungiform papillae count in smokers was 11.69 ± 5.06 per cm^2^, and in nonsmokers it was significantly (*p* < 0.0001) higher at 14.76 ± 4.55 per cm^2^ (Figure 7). A significant strong negative correlation was seen between smoking habit (packs/year) and fungiform papillae count (Figure 8). Additionally, a significant strong negative correlation of fungiform papillae count with age and BMI were noted in both smokers and nonsmokers (Figure 1, Figure 2, Figure 5, and Figure 6). 

The highest fungiform papillae count in the experimental group was recorded in a 25 year old female at 22.29 per cm^2^. Her smoking frequency was very low at 0.2 packs/year, and she had a BMI of 19.34. The lowest fungiform papillae count recorded was 1.68 per cm^2^ in a 42 year old male, with a smoking frequency of 30 packs/year and a BMI of 31.73. The former could taste PTC, while the latter could not. Among the nonsmokers, the minimum fungiform papillae count was 6.38 per cm^2^ in a 37 year old male with a BMI of 30.9 who could not taste PTC. On the contrary, the highest fungiform papillae count among the nonsmokers was 25.76 per cm^2^ in a 22 year old male with a BMI of 25.3 who was able to taste PTC. Another 28 year old male subject in the same group with a similar fungiform papillae count of 25.33 per cm^2^ and a BMI of 19.5 could not taste PTC.

### 3.4. PTC Taste Status

**Figure 9:**
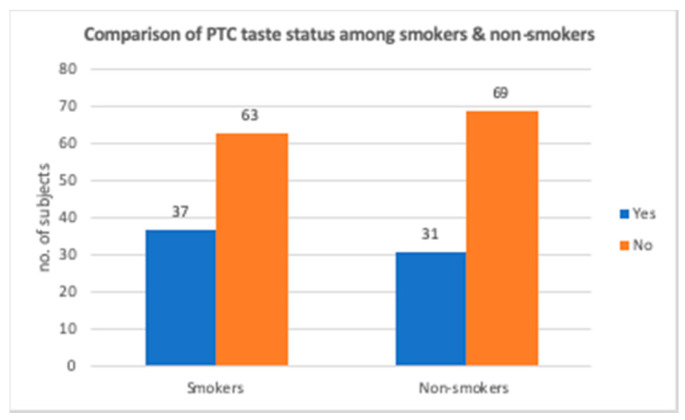
Comparison of PTC taste status among smokers and nonsmokers.

**Figure 10:**
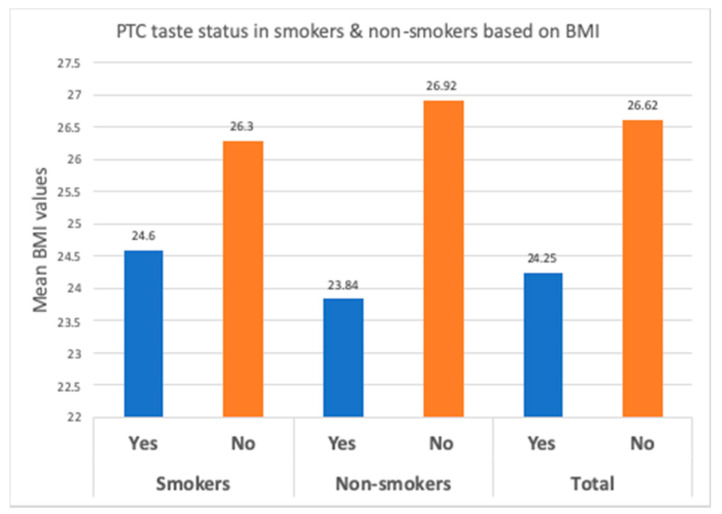
PTC tasting ability according to BMI in smokers and nonsmokers.

Out of 200 subjects, 37 smokers and 31 nonsmokers detected the bitterness from the taste strips (Figure 9). Smokers who could detect the taste had a fungiform papillae count of 14.32 ± 3.62 per cm^2^ (Table 3) and a BMI of 24.60 ± 3.18 (Table 2; Figure 10). Nonsmokers who could taste PTC had a fungiform papillae count of 17.50 ± 3.80 per cm^2^ (Table 3) and a BMI of 23.84 ± 3.01. (Table 2; Figure 10).

Smokers who positively responded to PTC had a significantly higher (*p* < 0.001) fungiform papillae count and a significantly lower (*p* = 0.0212) BMI (Table 2), as compared to smokers who could not respond to PTC. Nonsmokers who detected the PTC taste had a significantly higher (*p* < 0.001) fungiform papillae count and a significant lower (*p* < 0.001) BMI, as compared to nonsmokers who could not taste PTC (Table 3) (Figure 10).

The smokers who could not detect PTC taste showed a fungiform papillae count of 10.15 ± 5.16 per cm^2^ and a BMI of 26.3 ± 3.69. Nonsmokers who tested negative for PTC tasting had a fungiform papillae count of 13.54 ± 4.34 per cm^2^ and a BMI of 26.92 ± 3.01. The fungiform papillae count and BMI presented a significant difference in smokers and nonsmokers who could not detect PTC. Smokers who could not taste PTC had significantly fewer fungiform papillae (*p* < 0.001) and a significantly higher BMI (*p* = 0.0212) than the smokers who could taste PTC. A similar trend was seen among nonsmokers who showed a significantly lower number of fungiform papillae (*p* < 0.001) and significantly higher BMI values (*p* < 0.001) in subjects who gave a negative response to PTC.

## 4. Discussion

An age limit of 45 years was selected because past data suggest that there is a decline in the number of fungiform papillae as age progresses due to their degeneration [1,10]. In this study, an inverse relationship was found between age and fungiform papillae count. This trend was consistent in both smokers and nonsmokers. Increasing age not only affects taste function but also affects the lingual tactile sensitivity [22]. A similar trend of reduction in the number of fungiform papillae with age was also noticed among children [4]. This shows that increasing age with added factors such as smoking can be a cause for a depletion of fungiform papillae on the dorsum of the tongue, but smoking as a cause for the inability to taste PTC could not be established.

The results indicate that subjects with higher BMIs have a decreased number of fungiform papillae as compared to subjects with lower BMIs. This was true for both smokers and nonsmokers. Proserpio et al. investigated multiple aspects of obesity by comparing the taste threshold and the density of fungiform papillae in both normal-weight and obese subjects. Their results showed that obese subjects seem to have higher threshold values and a reduced number of fungiform papillae than normal-weight subjects [3]. These findings were also supported in studies by Mameli et al. [23] and Subash et al. [24]. Obese subjects seem to prefer energy dense food more than normal-weight subjects [3]. The smokers and nonsmokers who could taste PTC had a significantly lower BMI and a higher fungiform papillae count in comparison to those who could not detect PTC taste. On the contrary, a significantly higher BMI and a lower fungiform papillae count were found in subjects who could not taste PTC. This might indicate that a PTC tasting ability is not affected by smoking habits. Many authors have attributed a PTC and PROP (6-n-Propylthiouracil) tasting ability to supertaster phenomena. Supertasters have been linked to the presence of altered TAS2R genes, which enables them to detect the bitter taste of PTC and PROP [1,25]. 

In our study, there was a significant reduction in the numbers of fungiform papillae in smokers as compared to the nonsmokers. Similar findings were reported from studies that examined smoking in relation to fungiform papillae count [10,15,16]. This can be attributed to a detrimental effect on fungiform papillae from an assault of the myriad of harmful chemicals in cigarette smoke. Research data suggest that this is due to microvascular damage to the fungiform papillae, which leads to their degeneration [15,16]. A recent study described the overexpression of RNA transcripts for various inflammatory markers in the fungiform taste buds of obese human subjects. [26]. Kaufman et al. reported this to be a probable cause of their finding of reduced density of fungiform papillae and taste buds in college students who showed increased adipose tissue over a period of 4 years [27]. 

Although BMI is extremely clinically relevant, as it aids the primary care provider in cholesterol workup and management, diabetes screening, thyroid screening, and diet/exercise counseling, it still fails to depict an accurate level of body fat, which is the reason for obesity. For this reason, many new tools for assessing body composition based on bioelectrical impedance can provide an approximate, if not accurate, estimate of body fat [28]. Our results could not directly link decreased fungiform papillae count to obesity, as our subject sample did not show much of a variation in BMI. However, a definite relationship was established regarding increasing trends of BMI. However, further research can be performed using obese and normal-weight subjects as cases and controls, respectively. Additionally, our study sample had fewer female subjects, which was because of a lack of female smokers compared with male smokers.

## 5. Conclusions

Our study concludes that, as BMI increases, there is a lower likelihood of experiencing the bitter taste of PTC. Additionally, the ability to taste PTC decreases with diminishing numbers of fungiform papillae. Smoking does not affect bitter PTC tasting ability, despite negatively affecting the fungiform papillae count.

## Figures and Tables

**Table 1:** Correlation between fungiform papillae counts with age, smoking, and body mass index (BMI).

Group	Variables	*r*-Value	r^2^	*t*-Value	*p*-Value
Smokers	Age in years	−0.7843	0.6151	−12.5137	<0.001
	Pack-years	−0.8567	0.7340	−16.4437	<0.001
	BMI	−0.7252	0.5259	−10.4260	<0.001
Nonsmokers	Age in years	−0.8052	0.6483	−13.4420	<0.001
	Pack-years	-	-	-	-
	BMI	−0.6800	0.4624	−9.1816	<0.001

**Table 2:** Phenylthiocarbamide (PTC) taste status with according to BMI in smokers and nonsmokers.

Groups	Variable	PTC Taste Status	Mean BMI	SD	SE	*t*-Value	*p*-Value
Smokers	BMI	Yes	24.60	3.18	0.52	−2.3422	0.0212 *
		No	26.30	3.69	0.46		
Nonsmokers	BMI	Yes	23.84	3.01	0.54	−4.7225	<0.001
		No	26.92	3.01	0.36		
Total	BMI	Yes	24.25	3.11	0.38	−4.8533	<0.001
		No	26.62	3.35	0.29		

* *p* < 0.05.

**Table 3:** Comparison of PTC tasting status with FPAP scores in smokers, nonsmokers, and all subjects by the independent *t*-test.

Groups	Variable	PTC Taste Status	Mean FPAP	SD	SE	*t*-Value	*p*-Value
Smokers	FPAP	Yes	14.32	3.62	0.60	4.3327	<0.001
		No	10.15	5.16	0.65		
Nonsmokers	FPAP	Yes	17.50	3.80	0.68	4.3784	<0.001
		No	13.54	4.34	0.52		
Total	FPAP	Yes	15.77	4.01	0.49	5.4821	<0.001
		No	11.92	5.03	0.44		
